# Correction: SARS-CoV-2-specific immune response in COVID-19 convalescent individuals

**DOI:** 10.1038/s41392-021-00697-y

**Published:** 2021-07-30

**Authors:** Yunbao Pan, Xianghu Jiang, Liu Yang, Liangjun Chen, Xiaojiao Zeng, Guohong Liu, Yueting Tang, Chungen Qian, Xinming Wang, Fangming Cheng, Jun Lin, Xinghuan Wang, Yirong Li

**Affiliations:** 1 Department of Laboratory Medicine, Zhongnan Hospital of Wuhan University, Wuhan University, Wuhan, Hubei China; 2 Wuhan Research Center for Infectious Diseases and Cancer, Chinese Academy of Medical Sciences, Wuhan, China; 3 Department of Radiology, Zhongnan Hospital of Wuhan University, Wuhan University, Wuhan, China; 4 The Key Laboratory for Biomedical Photonics of MOE at Wuhan National Laboratory for Optoelectronics—Hubei Bioinformatics & Molecular Imaging Key Laboratory, Systems Biology Theme, Department of Biomedical Engineering, College of Life Science and Technology, Huazhong University of Science and Technology, Wuhan, Hubei China; 5 Reagent R&D Center, Autobio Diagnostics Co., Ltd, Zhengzhou, Henan China; 6 Reagent R&D Center, Shenzhen YHLO Biotech Co., Ltd, Shenzhen, Guangdong China; 7 Department of Gastroenterology, Zhongnan Hospital of Wuhan University, Wuhan University, Wuhan, China; 8 Center for Evidence-Based and Translational Medicine, Zhongnan Hospital of Wuhan University, Wuhan University, Wuhan, China; 9 Department of Urology, Zhongnan Hospital of Wuhan University, Wuhan University, Wuhan, China

**Keywords:** Infectious diseases, Immunological disorders

Correction to: *Signal Transduction and Targeted Therapy* 10.1038/s41392-021-00686-1, published online 7 July 2021

After online publication of the article^1^ the authors noticed an error in the figure legends of Fig. 1 and Supplementary Materials Fig. S2, where the “*P < 0.05, 0.05 < **P < 0.001, ***P < 0.001”. should be replaced by “*P < 0.05, **P < 0.01, ***P < 0.001”.

The original article has been corrected.
